# A Guide for Industrial Needleless Electrospinning of Synthetic and Hybrid Nanofibers

**DOI:** 10.3390/polym17223019

**Published:** 2025-11-13

**Authors:** Baturalp Yalcinkaya, Matej Buzgo

**Affiliations:** Respilon Membranes s.r.o., Nové sady 988/2, Staré Brno, 602 00 Brno, Czech Republic; m.buzgo@respilon.com

**Keywords:** industrial-scale nanofibers, nanoparticles, hybrid nanofibers, needleless electrospinning, filter, apparel, industrial applications

## Abstract

This study presents a comprehensive investigation into the large-scale production of synthetic and hybrid (nanoparticle-loaded) nanofibers using needleless electrospinning. A diverse range of polymers, including polyamide 6 (PA6) and its other polymer combinations, recycled PA6, polyamide 11 (PA11), polyamide 12 (PA12), polyvinyl butyral (PVB), polycaprolactone (PCL), polyacrylonitrile (PAN), polyvinylidene fluoride (PVDF), polyurethane (PU), polyvinyl alcohol (PVA), and cellulose acetate (CA), were utilized to fabricate nanofibers with tailored properties such as polymer solution concentrations and various solvent systems. Furthermore, an extensive variety of nano- and micro-particles, including TiO_2_, ZnO, MgO, CuO, Ag, graphene oxide, CeO_2_, Er_2_O_3_, WO_3_, MnO_2_, and hyperbranched polymers, were incorporated into the polymeric systems to engineer multifunctional nanofibers with enhanced structural characteristics. The study examines the impact of polymer–nano/micro-particle interactions, fiber morphology, and the feasibility of large-scale production via needleless electrospinning. The resulting nanofibers exhibited diameters starting from 80 nm, depending on the polymer and processing conditions. The incorporation of TiO_2_, CeO_2_, WO_3_, Ag, and ZnO nanoparticles into 15% PA6 solutions yielded well-dispersed hybrid nanofibers. By providing insights into polymer selection, nano- and micro-particle integration, and large-scale production techniques, this work establishes a versatile platform for scalable hybrid nanofiber fabrication, paving the way for innovative applications in nanotechnology and materials science.

## 1. Introduction

Electrospinning has undergone a significant transformation, advancing from laboratory-scale production to industrial pilot and large-scale manufacturing within a quarter of the 21st century [1,2,3,4,5]. One of the first studies on electrospinning was conducted in the United States by Formhals [6], who demonstrated the first laboratory-scale electrospinning prototypes. Later, in the USSR, Petryanov [7] advanced the process to semi-industrial fiber production. One of the most pivotal innovations in this progress was the development of the needleless electrospinning method, pioneered by Oldřich Jirsák [8]. Instead of using a needle apparatus, this technique utilizes a conductive cylinder partially immersed in a polymer solution. As the cylinder rotates around its axis, it carries the polymer solution, forming a thin film layer on its surface. When a high-voltage electric field is applied, the surface tension of the polymer solution on the cylinder is overcome, leading to the formation of multiple Taylor cones and spinning jets [9]. Thanks to this breakthrough, electrospinning technology has reached a new era in nanofiber production.

This advancement paved the way for high-efficiency needleless electrospinning systems, leading to the establishment of commercial enterprises, with Elmarco s.r.o [10,11,12] (Czech Republic) being one of the pioneers in the field. The contributions of these innovative companies have significantly impacted nanotechnology, enabling the development of various high-performance functional products across different industries [13,14,15,16]. Among the most beneficial applications of nanofibers are air filtration systems [17,18]. The extremely fine structure of nanofibers results in a large specific surface area, high porosity, and small pore sizes. This unique morphology enables nanofiber-based filters to perform surface filtration efficiently, achieving a removal rate of up to 99.97% for PM0.3 without clogging, thereby ensuring easy cleaning and maintaining high air permeability, such as 85 L/m [19,20,21]. Another significant market shift occurred with the decision to ban the use of polytetrafluoroethylene (PTFE) polymers in various industries. As a result, thermoplastic polyurethane (TPU) nanofibers, known for their high air permeability (62 mm/s) and water repellency with a water contact angle of 151.2°, have gained substantial demand, particularly in the functional apparel industry [22,23,24,25]. Due to their elastic and durable structure, PU nanofibers have demonstrated twice the water vapor permeability compared to PTFE-based membranes while maintaining the same level of water resistance [26].

With each advancement in electrospinning technology, polymers commonly used in the plastics and textile industries have been gradually processed into a nanofiber form [27]. Regardless of the production method or system, both synthetic and hybrid polymers have found their place at the nanoscale [28]. Among the most frequently electrospun and widely utilized nanofibers are synthetic polymers such as polyamide [29], polyurethane [24], and polyacrylonitrile [30]. In addition to the high-volume production of synthetic polymers, various other polymers have been extensively studied and fabricated in laboratory settings [31,32]. These include polyvinyl alcohol (PVA) [33], polyvinylpyrrolidone (PVP) [34], polycaprolactone (PCL) [35], polylactic acid (PLA) [36], polyglycolic acid (PGA) [37], polyethylene oxide (PEO) [38], polyethylene terephthalate (PET) [39], polybenzimidazole (PBI) [40], poly (amide-imide) (PAI) [41], polycarbonate (PC) [42], polyvinylidene fluoride (PVDF) [43], polyaniline (PANI) [44], and polypyrrole (PPy) [45]. These polymers continue to be widely explored for their potential applications in electrospinning.

Natural polymers that have been electrospun into nanofiber form include collagen [46], gelatin [47], chitosan [48], cellulose and its derivatives [49], alginate [50], silk fibroin [51], hyaluronic acid [52], dextran [53], and keratin and fibrinogen [54]. Although these materials are primarily utilized in laboratory settings or small-scale applications, polymers such as polyamide 6 [55], polyurethane [56,57], polyacrylonitrile [58], and collagen [59] have found extensive use in the various industry, leading to large-scale production and commercialization.

Upon realizing the evidence that natural and synthetic nanofibers alone could not achieve the desired high functionality, researchers began developing composite, blend, and hybrid nanofibers. By combining different materials, nanofibers with enhanced properties, including antibacterial, antiviral, waterproof, conductive, heat-resistant, photocatalytic, and piezoelectric functionalities, were successfully created. Notable examples include PLA/PCL [60,61,62], PCL/chitosan [63,64,65], PVA/gelatin [66,67], PAN/PANI [68,69], PVP/TiO_2_ [70,71], PVA/GO [72], and PVDF/carbon nanotubes [73], each offering specific advanced characteristics tailored for diverse applications.

Only a limited number of polymers are suitable for the free-surface nanofiber electrospinning system. Achieving optimal fiber production characterized by fine fiber morphology, bead-free structure, and the absence of non-fibrous areas requires extensive research and development. The optimization process is primarily influenced by factors such as polymer solution concentration, additives, and process parameters, including the distance between electrodes and the applied voltage.

In this study, we hypothesize that each polymer possesses a specific concentration range that enables the formation of uniform, bead-free nanofibers under optimized large-scale production conditions using the needleless electrospinning method. Therefore, the primary purpose of this work is to systematically investigate and optimize a wide range of polymer and their nanoparticle-loaded solution suitable for industrial-scale electrospinning. By evaluating the influence of polymer, solvent, and nanoparticle type and concentration on fiber morphology and spinnability, the study aims to establish a comprehensive guideline for optimal parameters in large-scale nanofiber fabrication. The findings are expected to serve as a practical reference for both academic researchers and industrial manufacturers in developing high-performance and functional electrospun nanofiber products, such as water-air separation membranes, biomedical and healthcare applications, and antibacterial and breathable waterproof membranes.

## 2. Materials and Methods

### 2.1. Materials

Polyamide 6 (PA6), regenerated PA6 (r-PA6), polyamide 11 (PA11), polyamide 12 (PA12), polyvinyl butyral (PVB), polycaprolactone (PCL), polyacrylonitrile (PAN), polyvinylidene fluoride (PVDF), polyurethane (PU), polyvinyl alcohol (PVA), cellulose acetate (CA) and chitosan (CS) were chosen as polymeric source for electrospinning. Nanoparticles, including titanium dioxide (TiO_2_), zinc oxide (ZnO), magnesium oxide (MgO), copper oxide (CuO), silver (Ag), cerium oxide (CeO_2_), erbium oxide (Er_2_O_3_), tungsten trioxide (WO_3_), manganese dioxide (MnO_2_), graphene oxide (GO), and hyperbranched polymers were used as additive. The complete list of polymers, including their commercial names, characteristics, and suppliers, is presented in Table 1. The types of nano- and micro-particles, their sizes, and origins are presented in Table 2. Table 3 presents the list of solvents along with their sources.

### 2.2. Solution Preparation

Two distinct steps have been employed to prepare solutions of synthetic polymers and their hybrid formations. The initial portion of the synthetic polymer powder/granule is introduced to solvents while gently stirring to prevent the formation of aggregates. The solutions are stirred overnight to guarantee total dissolution and uniformity. Hybrid solution preparation involves the gradual incorporation of metal oxide particles into the solvent system, followed by thorough mixing with an IKA T-25 ULTRA-TURRAX homogenizer from IKA Werke GmbH, Staufen im Breisgau, Germany, for 30 min. Once an optimal dispersion of micro/nano particles is achieved within the solvent system, the micro/nano particles are introduced into the polymer solution at the concentration specified in the provided concentration table (refer to the respective polymer solution concentration table in the relevant polymer section) and further mixed mechanically. Each polymer solution was prepared in 50 mL bottles.

### 2.3. Electrospinning of Synthetic and Hybrid Nanofibers

**Figure 1:**
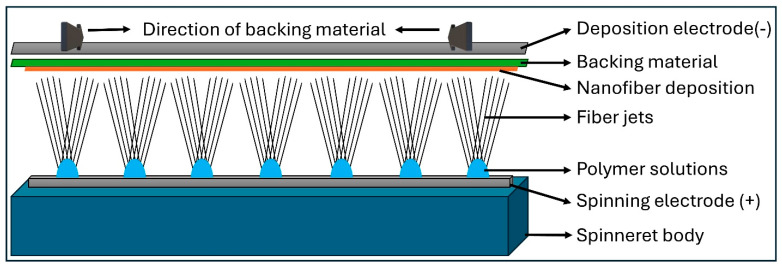
Needleless electrospinning spinneret body and spinning process.

Nanofibers were produced using a free-surface needleless electrospinning technique, in which the polymer solution was supplied onto a thin, fixed conductive metal spinneret Figure 1.

**Figure 2:**
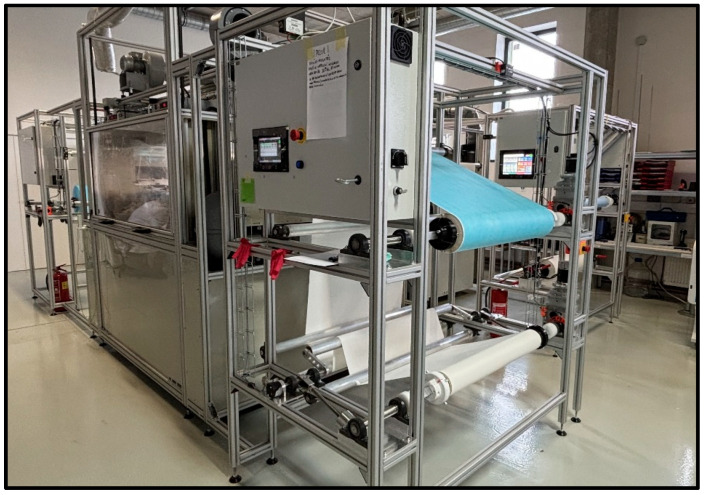
Industrial-size needleless electrospinning devices.

The electrospinning system utilized in this study is an industrial-scale machine with a width of 80 cm and a length of 2 m. Before nanofiber production, it is essential to maintain the electrospinning chamber at specific humidity and temperature levels. These environmental conditions are regulated by an integrated air-drying system, which controls relative humidity and temperature to ensure stable fiber formation. The industrial-scale needleless electrospinning setup operates with a roll-to-roll nanofiber deposition system Figure 2.

A 45 gsm polypropylene nonwoven substrate is loaded into the unwinder unit and fed into the spinning chamber, where nanofibers are deposited onto the backing material. The rewinder unit then collects the coated nonwoven. All other process parameters, including polymer solution properties and electrospinning conditions, are optimized for each polymer type and detailed in the respective nanofiber sections.

### 2.4. Characterization of Nanofibers

The morphology and average diameter of the produced synthetic and hybrid nanofibers were analyzed using scanning electron microscopy (SEM, Phenom ProX, Thermo Fisher Scientific, Waltham, MA, USA). To prepare the samples, nanofiber specimens with their nonwoven substrate were cut into appropriate dimensions (0.75 cm^2^) to ensure compatibility with the SEM sample holder. Since nanofibers on a nonwoven substrate are typically non-conductive, a conductive coating is necessary to minimize charging effects and enhance imaging quality. Therefore, a 5 nm gold layer was deposited onto the samples using a LUXOR_AU sputter coater (Aptco Technologies, Frankfurt am Main, Germany), ensuring optimal conductivity for high-resolution SEM imaging.

## 3. Results and Discussion

### 3.1. Electrospinning of CA Nanofibers

The process of cellulose acetate is essential due to its unique properties and prospective uses in various industries [74]. The most significant aspects are its hydrophilicity, biodegradability, and sustainability. However, the process of electrospinning cellulose acetate polymer into nanofibers is a complex process. There are several types of cellulose acetate (CA), including cellulose acetate butyrate (CAB) and cellulose acetate propionate (CAP). Moreover, cellulose acetate from Eastman is produced by the esterification of cellulose with differing degrees of substitution. The classification of these compounds as diacetate or triacetate is contingent upon their level of acetylation. Cellulose acetates, such as CA-320S, possess a high hydroxyl content, indicating that not all hydroxyl groups on the cellulose backbone are subjected to acetylation. This signifies that certain goods are not completely acetylated (not triacetate) but fall within the diacetate category. CAB and CAP products are mixed esters, including acetate and other ester groups (such as butyrate or propionate). Hence, they are not distinctly categorized as diacetate or triacetate [75,76,77]. An important fact is that not all cellulose acetate polymers can be spun. After conducting an extensive analysis of several types of cellulose acetate and grades (product name indicates the solution viscosity, which correlates with molecular weight 0.2–0.5–1–3–5–10–30) [78], CA 398-10 type CA was utilized for the manufacturing of nanofibers. The process parameters of CA nanofiber are given in Table 4. The polymer concentration, mixture of the solvents, solvent ratios, fiber diameter, and SEM images are given in Table 5.

The solution feed rates are typically expressed in mbar/h in industrial-size electrospinning devices; however, a direct calculation of the flow rate in mL/h is also possible. Each polymer solution was prepared in 50 mL, and each production was set at 30 min.

Flow Rate = Total Volume/Time = 50 mL/0.5 h =100 mL/h

Cellulose acetate can be easily transformed into nanofibers using a DMAC/Acetone [79] solvent system due to the excellent balance of solubility, viscosity control, and evaporation behavior provided by this mixture. DMAC acts as a suitable solvent for CA, ensuring sufficient polymer chain entanglement and enhancing solution conductivity, which is essential for stable electrospinning [80]. Meanwhile, acetone’s high volatility enables rapid solvent evaporation and fiber solidification during the spinning process. Multiple concentrations, grades, and solvent systems were examined, and it was found that just one concentration and solvent ratio (15%, 5:5) successfully enabled the electrospinning of cellulose acetate polymer solutions. Nevertheless, the nanofibers created are not sufficiently strong or stable on the surface of the substrate. It is advisable to combine the CA polymer with another polymer for improved results.

### 3.2. Electrospinning of PCL Nanofibers

Polycaprolactone (PCL) is a biodegradable polymer, a type of polyester that the human body can readily absorb [81]. It is commonly employed in various medical fields, including drug administration, tissue engineering, and wound healing [82]. PCL is renowned for its excellent biocompatibility, low toxicity, and effortless processing [83]. The process parameters for PCL nanofibers are listed in Table 6. The polymer concentration, solvent mixture, solvent ratios, fiber diameter, and SEM images are presented in Table 7.

Polycaprolactone is a hydrophobic, semi-crystalline polymer that requires an appropriate solvent system for effective electrospinning [84]. A solvent mixture of acetic acid, formic acid, and chloroform works well [85]. On the other hand, chloroform provides good solubility for the hydrophobic PCL chains, enabling fast solvent evaporation and facilitating fiber solidification. Similarly, a well, while ethanol adjusts the solution’s polarity and conductivity, leading to better fiber formation and reducing defects such as beads [86]. These mixed solvent systems strike a balance between solubility, viscosity, conductivity, and evaporation rate, all of which are critical factors for producing uniform and smooth PCL nanofibers.

The electrospinning of PCL using chloroform as a solvent yielded non-uniform nanofibers, rather than uniform nanofibers. Alternative solvent systems and polymer blending strategies were explored to address this issue. The use of acidic solvent solutions significantly improved the quality and morphology of PCL nanofibers while also enhancing production efficiency. Furthermore, incorporating natural polymers such as cellulose acetate and chitosan, as well as synthetic polymers like polyethylene oxide, in the blending process further increased fiber uniformity and productivity. Although microfibers lack the high surface area and nanoscale features of nanofibers, they can still be advantageous for tissue engineering and medical applications. Their larger diameters provide increased mechanical strength and structural integrity, which support cell adhesion, proliferation, and extracellular matrix deposition, making them suitable for use as scaffolds in regenerative medicine.

### 3.3. Electrospinning of PA6 Nanofibers

Polyamide 6 is a widely used polymer in the production of nanofibers, particularly in the filtration and apparel industries [87]. It is a market leader in these segments due to its excellent mechanical properties and versatility. Coating corrugated 80/20 cellulose/synthetic paper with PA6 nanofibers enhances filtration efficiency, enabling it to meet F-class or HEPA (High-Efficiency Particulate Air) standards [88]. Additionally, PA6 nanofibers are utilized in high-performance sportswear [23,89] and personal protective clothing [90] due to their breathability, durability, and lightweight nature. Therefore, the development and characterization of PA6 nanofibers using an industrial-scale electrospinning device are essential for optimizing their performance in these applications. The process parameters of PA6 nanofiber are given in Table 8. Various concentrations and diverse solvent systems have been investigated, and the results are presented in Table 9.

Polyamide 6 (PA6) nanofibers can be effectively fabricated by electrospinning using solvent systems such as acetic acid (AA) and formic acid (FA) with chloroform or dichloromethane (DCM) [91]. The combination of AA and FA provides good solubility for PA6 due to their polar protic nature, where formic acid enhances solubility and acetic acid helps control viscosity and fiber smoothness [92]. The addition of chloroform, a volatile, non-polar solvent, can improve fiber formation by reducing surface tension and promoting faster solvent evaporation, resulting in smoother fibers with potential surface porosity if used in excess. Alternatively, replacing chloroform with dichloromethane (DCM), a more volatile solvent, further increases the evaporation rate during spinning. Various solvent and co-solvent systems combinations, along with two different PA6 sources, were investigated to generate a dataset for evaluating the morphology of PA6 nanofibrous membranes. All polymer solutions produced high-quality nanofibers free from beads and non-fibrous regions, except those with low polymer concentrations. Blending solvent and co-solvent systems proved highly efficient in facilitating uniform nanofiber deposition. Characterization of morphology revealed exceptionally low fiber diameter values, which are highly beneficial for filtration applications. The measured fiber diameters ranged between 100 and 250 nm, falling within the optimal range for high-performance filtration. Evaluation tests confirmed that PA6 is one of the most suitable polymers for industrial-scale nanofiber production using needleless electrospinning technology. Low-diameter nanofibers are ideal for air and water filtration due to their high surface area and superior filtering capacity. In contrast, larger-diameter fibers provide enhanced mechanical strength, making them well-suited for apparel membranes.

### 3.4. Electrospinning of PA11 and PA12 Nanofibers

Polyamide 11 and Polyamide 12 are polymers that exhibit exceptional resistance to chemicals, heat, and mechanical stress, surpassing even the performance of Polyamide 6 [93]. PA11 and PA12 have not been previously subjected to electrospinning in industrial-scale equipment. Preparing their polymer solution is as challenging as compared to PA6 [94]. In this research, we attempted to prepare nanofibers on a large scale using PA11 and PA12 for the first time. The process parameters of PA11 and PA12 nanofibers are given in Table 10. Various concentrations and diverse solvent systems have been studied, and the results are illustrated in Table 11.

The process of producing nanofibers from Polyamide 11 and Polyamide 12 is challenging. Although the fibers, ranging in size from nano to a few microns, were successfully deposited, their characteristics, production rates, and surface structures did not meet the intended standards. This approach has demonstrated the potential for further enhancement. The combination of polyamide 11 and polyamide 12 with polyvinyl butyral resulted in the formation of dense nanofibers, simultaneously enhancing both production and quality.

### 3.5. Electrospinning of PAN Nanofibers

Polyacrylonitrile is a synthetic polymer that is widely used in various industrial applications [95]. It is a thermoplastic polymer that is made from acrylonitrile monomers through polymerization [96]. The polymer has a linear structure, consisting of repeating units of acrylonitrile. PAN polymer electrospinning is particularly useful for creating nanofibers with diameters ranging from 50 to 300 nanometers [97]. These fibers have a high surface area, high porosity, and high mechanical strength, making them ideal for various applications, such as filtration. The process parameters of the PAN nanofiber are given in Table 12. Different concentrations and solvent systems have been studied, and the results are illustrated in Table 13.

Polyacrylonitrile nanofibers are widely fabricated by electrospinning using solvents like N, N-dimethylformamide and N, N-dimethylacetamide, both of which offer good solubility for PAN due to their strong polar aprotic nature [98]. DMF is the most preferred solvent because of its balanced volatility, high dielectric constant, and ability to produce uniform, smooth, and fine nanofibers due to stable jet formation and controlled solvent evaporation [99]. In contrast, DMAC has a slightly higher boiling point and slower evaporation rate, which can result in thicker fibers, wet deposition, or fused structures if spinning conditions are not optimized. The slower evaporation of DMAC may require higher applied voltage, longer collector distance, or lower flow rates to ensure complete solvent removal [100]. Polyacrylonitrile polymers have exceptional thermal and chemical resilience, making them very suitable for nanofiber applications [101].

Additionally, they offer versatile nanofiber production capabilities when used in electrospinning technology. PAN exhibits solubility in several aromatic polymers, with DMAC and DMF being particularly favorable choices due to their ability to produce high-quality and productive nanofibers. In this set of samples, PAN was successfully electrospun using an industrial-scale electrospinning apparatus. Increasing concentration resulted in the formation of high-quality surface morphologies without the presence of beads. PAN dissolved in DMF solvent has a higher nanofiber deposition efficiency due to the lower boiling point. However, DMAC solvent can also serve as a viable alternative for polyacrylonitrile polymers.

### 3.6. Electrospinning of PVDF Nanofibers

Polyvinylidene fluoride (PVDF) is a widely utilized polymer in large-scale industrial production for creating nanofibers [102]. This is primarily due to its ability to be electrospun, as well as its chemical and mechanical stability, piezoelectric characteristics, and ease of processing [103]. Exceptional features suggest that the electrospinning of PVDF polymer holds excellent promise as a viable method. The process parameters of PVDF nanofiber are given in Table 14. Various solution concentrations have been studied, and the results are illustrated in Table 15.

The use of industrial-scale needleless electrospinning for polyvinylidene fluoride has demonstrated the feasibility of producing nanofibers. Nevertheless, there is still potential for enhancing the quality of PVDF nanofibers. Nanofibers with minimal bead formation and a low non-fibrous region were successfully produced utilizing a concentration of only 20% *w*/*v*. Hence, to enhance the quality of PVDF nanofibers, it is necessary to adjust both environmental and process parameters. This is because PVDF is a highly sensitive polymer to humidity in electrospinning technology [104,105]. Therefore, precise calibration is crucial for achieving an improved nanofiber morphology.

### 3.7. Electrospinning of PU Nanofibers

The unique features and numerous applications of electrospun polyurethane nanofibers have made them highly important in various sectors [106]. The most essential characteristics of polyurethane nanofibers are their customizable mechanical strength and their ability to act as barriers against unwanted liquids and particles [107,108]. The process parameters for the production of PU nanofibers are listed in Table 16. Various concentration systems have been studied, and the results are illustrated in Table 17.

The electrospinning of polyurethane polymers is in great demand for the production of nanofibers because of their exceptional mechanical strength, impact resistance, and flexibility. PU nanofiber is an ideal choice for textile garments or coveralls. Due to their simplicity in lamination, polyurethane nanofibers can be effortlessly combined with other textile surfaces, such as knitted or woven fabrics made of polyester or cotton. The nanofibers become thicker and bead-free as the concentration of PU increases.

### 3.8. Electrospinning of PVB Nanofibers

PVB is a synthetic polymer classified as a member of the polyvinyl acetal resin group [109]. Nevertheless, the electrospinning process enables the fabrication of nanofibers possessing distinctive properties and promising applications [110]. Primarily, it exhibits solubility in nearly all solvents commonly employed in electrospinning, particularly those that are non-toxic. Consequently, this attribute results in PVB nanofibers being highly environmentally friendly [111]. It can also be mixed with other polymers because it is soluble in different solvents. The process parameters of the PVB nanofiber are given in Table 18. Various concentrations and diverse solvent systems have been studied, and the results are illustrated in Table 19.

The utilization of a solvent and co-solvent combination including ethanol and chloroform for dissolving PVB is crucial due to the inherent high flammability of ethanol. Additionally, the electrospinning process occasionally generates sparks between the lower and top electrodes. In order to address safety concerns and enhance the efficiency of PVB nanofiber production, solvent solutions are employed. Among these, chloroforms have been found to yield high-quality fibers, but with a thicker consistency. The quality of nanofibers obtained from ethanol with ethylene acetate and acid systems was not superior to that of nanofibers obtained from chloroform systems. The microscope images reveal the production of significantly thick fibers using needleless electrospinning, indicating the potential for further enhancement.

### 3.9. Electrospinning of PVA Nanofibers

The electrospinning process is commonly employed to fabricate nanofibers with diameters ranging from a few nanometers to micrometers using polyvinyl alcohol (PVA) [107]. PVA and PVB are synthetic polymers classified under the polyvinyl ester group, each possessing unique characteristics and uses. PVA is a water-soluble polymer that is generated by partially or completely hydrolyzing polyvinyl acetate. Hence, it is necessary to use a crosslinking treatment or agent either prior to or following the electrospinning process of PVA [108]. The process parameters of the PVA nanofiber are given in Table 20. Various concentrations and diverse solvent systems have been studied, and the results are illustrated in Table 21.

The large-scale fabrication of polyvinyl alcohol nanofibers can be simply accomplished by utilizing industrial-sized needleless electrospinning. PVA polymer was spun into nano or microfibers using various concentrations and solvent solutions.

### 3.10. Electrospinning of PA6 Nanofibers Containing Nanoparticles

Polyamide 6 polymer is an excellent candidate for filtering applications and textile manufacturing. Consequently, the incorporation of nanoparticles holds significant potential to enhance PA6 nanofibers by enabling functionalities such as self-cleaning, photocatalysis, chemical absorption, and antibacterial and antiviral properties.

This comprehensive investigation involved 15 wt% PA6 dissolved in the AA/FA/CH solution system, which was mixed with various nanoparticles at concentrations of 5, 15, and 30 wt%. The tables below display the SEM pictures of the utilized nanoparticles and their concentrations in relation to Polyamide 6 nanofibers. The process parameters of PA6 hybrid nanofibers are given in Table 22. Various concentrations and diverse nanoparticle systems have been studied, and the results are illustrated in Table 23.

Polyamide 6 solutions blended with nanoparticles (as shown in the Table above) were successfully spun into hybrid nanofibers using an industrial-size needleless electrospinning method. Through the implementation of large-scale production, we have successfully manufactured a quantity of hybrid nanofiber fabric measuring 3–4 m^2^ from each polymer solution.

The SEM images indicate that the nanofibers possess well-defined morphologies, with little to no presence of beads. Nanofiber mats have no non-fibrous area and an extremely small fiber diameter. The distribution of fiber sizes ranges from 80 nm to 300 nm. The polyamide 6 nanofibers possess a thin structure, resulting in nanoparticles that appear to be primarily situated on the surface of the fibers. Elevating the concentration of nanoparticles in polymer solutions undoubtedly enhances nanoparticle loading on the surface of the nanofibers.

**Figure 3:**
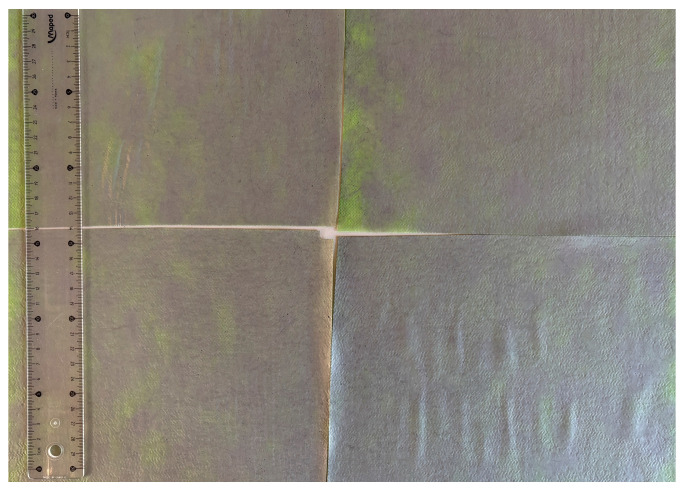
PA6 nanofibers with GO nanoparticles.

In this study, a new method for achieving uniform dispersion using the IKA T-25 ULTRA-TURRAX, IKA-Werke, Staufen, Germany, a high-shear disperser (or homogenizer), was attempted. Its main advantage lies in generating extremely high shear forces (up to 24,000 rpm), which can break apart aggregates by applying intense mechanical shear. This method worked well for some metal oxides, even at higher concentrations; however, certain metal oxides still exhibited aggregation. This may be attributed to free-surface electrospinning, as the amount of polymer solution used in industrial-scale electrospinning is always large. The surface of the fibers is well scattered with metal oxide nanoparticles, specifically TiO_2_, ZnO, CeO_2_, WO_3_, silver, and combinations of TiO_2_ and ZnO particles. MgO, CuO, and Er_2_O_3_ tend to agglomerate and form clusters of nanoparticles on the surface of nanofibers. Graphene nanoparticles are not visible when observed under a scanning electron microscope. However, the nanofibrous fabric exhibits a black coloration on its surface due to the presence of graphene (Figure 3).

### 3.11. Electrospinning of PAN Nanofibers Containing Nanoparticles

Polyacrylonitrile is a highly promising polymer for the large-scale production of nanofibers. In this section, a hybrid nanofiber utilizing polyacrylonitrile polymers and nanoparticles has been developed due to its adaptable manufacturing and effortless spinnability. The process parameters of PAN hybrid nanofibers are given in Table 24. Various concentrations and diverse nanoparticle systems have been studied, and the results are illustrated in Table 25.

### 3.12. Electrospinning of PU Nanofibers Containing Nanoparticles

The polyurethane polymer has been selected as the third most promising type of polymer for creating hybrid nanofibers. These nanofibers are generated by adding nanoparticles to the polyurethane nanofiber matrices, which enhances the material’s capabilities. The process of hybridization enables the combination of distinct characteristics from both nanofibers and nanoparticles, resulting in customized materials that exhibit improved performance. The strong mechanical strength and flexible structure of polyurethane hybrid nanofibers make them highly promising for applications in apparel and garment textiles. Consequently, a selection of nanoparticles was mixed with polyurethane nanofibers in this series of experiments. The process parameters of PU hybrid nanofibers are given in Table 26. Various concentrations and diverse nanoparticle systems have been studied, and the results are illustrated in Table 27.

The interaction of polyurethane hybrid nanofibers with metal oxide nanoparticles has been successfully achieved on a large scale using industrial-scale needleless electrospinning. Each 50 mL polymer solution yielded approximately 5–6 square meters of hybrid nanofibers, with a thickness range of 8 to 20 microns. The morphology and quality of the nanofibers were excellent, with their thickness ranging from 350 nm to 550 nm. Consequently, specific nanoparticles were found both on the surface and within the nanofiber matrices. These behaviors are observable using TiO_2_, WO_3_, and ZnO nanoparticles. Graphene nanoparticles were not visible when observed under a scanning electron microscope. However, the nanofibrous fabric exhibited a black coloration on its surface due to the presence of graphene. Elevating the concentration of nanoparticles in polymer solutions undoubtedly enhances nanoparticle loading on the surface of the nanofibers. The phenomenon can be readily analyzed using scanning electron microscopy images.

The development of pristine and nanoparticle-loaded nanofibers produced via industrial-scale electrospinning represents a significant advancement in materials science and nanotechnology. In Table 28, the exploration of pristine and nanoparticled nanofibers in industrial electrospinning processes was summarized and described.

## 4. Conclusions

In the present study, a diverse range of widely utilized polymers and nanoparticles were successfully processed into pristine and hybrid (nanoparticle-loaded) nanofibers using needleless electrospinning. Critical processing parameters and polymer solution preparation strategies were systematically investigated and reported. Optimal nanofiber morphologies of PA6, PA11, PA12, PVB, PCL, PAN, PVDF, PVA, PU, and CA were identified based on scanning electron microscopy analysis, and indicated with a star symbol (★) to assist future studies. The resulting nanofibers exhibited diameters starting from 80 nm. Additionally, potential application areas for the produced nanofibers were outlined.

CA (430 nm), PA11 (4590 nm), and PA12 (790 nm) generally formed nano- and micro-structured fibers, resulting in mechanically weak, cotton-like fibers. PVA (245 nm), PVB (720 nm), and PVDF (495 nm) fibers, on the other hand, produced durable and fine nanofibers with very high production efficiency. PCL fibers formed strong nano- and micro-fibers (700–3000 nm), which can be used in applications such as biodegradable materials for wound dressings, providing robust and reliable fiber structures. Specifically, optimal electrospinning parameters were determined for the commonly studied PA6, PAN, and PU nanofibers. Highly uniform and stable PA6 nanofibers with an average diameter of approximately 250 nm were obtained from 12.5% and 15% PA6 solutions using AA/FA/DCM and AA/FA/CH solvent systems. The incorporation of TiO_2_, CeO_2_, WO_3_, Ag, and ZnO nanoparticles into 15% PA6 solutions yielded well-dispersed hybrid nanofibers. Furthermore, PAN and PU nanofibers produced from 15% polymer solutions in DMF exhibited relatively larger diameters, enhancing both surface attachment and encapsulation of nanoparticles. The introduced microparticles predominantly resided on the nanofiber surfaces, maintaining their functional activity.

The central hypothesis of this work was that a unified process–structure relationship can be established for a broad spectrum of polymers and hybrid nanofibers through needleless electrospinning, enabling reproducible large-scale production without the need for material-specific redesign. The present results confirm this hypothesis and provide the first experimentally validated guideline integrating polymer type, solvent system, and operational parameters for scalable electrospinning. In conclusion, the findings of this study provide a comprehensive guideline for the production and optimization of pristine and hybrid nanofibers using needleless electrospinning. The presented results are expected to serve as a valuable resource for researchers, facilitating future developments in nanofiber fabrication and broadening their potential applications across various industrial sectors.

Moving forward, future research is anticipated to focus on expanding the library of processable polymers and functional nanoparticles compatible with needleless electrospinning. The integration of machine learning and artificial intelligence-assisted optimization techniques could significantly accelerate the parameter screening process, reducing experimental workload and improving reproducibility. Moreover, the development of environmentally friendly solvent systems and bio-based polymers will be crucial to align nanofiber production with sustainability goals. The exploration of multifunctional nanofibers tailored for emerging fields, such as smart textiles, energy harvesting, environmental remediation, and biomedical applications, is expected to further enhance the industrial relevance and societal impact of nanofiber technologies.

## Figures and Tables

**Table 1:** The list of polymers.

Polymer Name	Commercial Name	Type and Key Properties	Supplier Name	Origin Country
CA	Cellulose acetate (CA-398-10)	Semi-synthetic polymer derived from cellulose; 39.8% acetyl content; Tg ≈ 190 °C;	Eastman	Kingsport, TN, USA
PCL	Polycaprolactone (Mn) 80,000	Aliphatic biodegradable polyester; low Tg (−60 °C), Tm ≈ 60 °C; high flexibility.	Sigma Aldrich	Burlington, MA, USA
PA6	Ultramid^®^ B24	Synthetic polyamide with high crystallinity, Tg ≈ 50 °C, Tm ≈ 220 °C; good spinnability and mechanical strength	BASF	Ludwigshafen, Germany
PA6	Econyl 27	Chemically regenerated nylon 6 from waste; same structure as PA6 but higher purity; sustainable alternative	Econyl	Trento, Italy
PA11	Rilsan^®^	Long-chain aliphatic bio-polyamide from castor oil; Tm ≈ 190 °C; low moisture uptake, high flexibility	Arkema	France, Colombes
PA12	VESTAMID^®^ L	Long-chain polyamide; Tm ≈ 178 °C; very low water absorption, high chemical resistance.	Evonik	Germany, Essen
PVB	Mowital B 60 H	Amorphous thermoplastic with hydroxyl groups (~18–22%); Tg ≈ 70 °C.	Kuraray	Germany, Hattersheim
PAN	Polyacrylonitrile	Semi-crystalline polymer; Tg ≈ 95 °C; precursor for carbon fibers; highly polar nitrile groups aid jet stability	Goodfellow	Huntingdon, UK
PVDF	Kynar 761A	Semi-crystalline fluoropolymer; Tm ≈ 170 °C; piezoelectric and hydrophobic; excellent chemical resistance	Arkema	Colombes, France
PU	Larithane AL 286	Thermoplastic elastomer; Shore A ≈ 85; soft segment polyester-based; high elasticity, durable	Novotex	Gaggiano, Italy
PVA	Poval™ 5-88	Water-soluble synthetic polymer; 88 mol% hydrolyzed; Mw ≈ 89,000–98,000.	Kuraray	Hattersheim, Germany
CS	Chitosan—Medium molecular weight	Natural cationic polysaccharide; deacetylation ~75–85%; Mw ≈ 190–310 kDa.	Sigma Aldrich	Burlington, MA, USA

**Table 2:** List of additives.

Additive	Particle Size	Supplier Name
TiO_2_	200 nm	Nanografi (Düsseldorf, Germany)
ZnO NP	30–50 nm	Nanografi
MgO NP	55 nm	Nanografi
MgO NP	500–1500 nm	Nanografi
CuO NP	38 nm	Nanografi
CuO NP	78 nm	Nanografi
CuO NP	20 µm	Argaman (Jerusalem, Israel)
Ag	100 nm	Nanografi
Graphene Oxide	<2 µm	Graphene-XT (Bologna, Italy)
CeO_2_	8–28 nm	Nanografi
Er_2_O_3_	8–90 nm	Nanografi
WO_3_	55 nm	Nanografi
MnO_2_	<200 mesh size (micron powder)	Nanografi
Hyperbranched Polymer	PFLDHB-G4-PEG10K-OH	Polymer Factory (Stockholm, Sweden)
Hyperbranched Polymer	PFLDHB-G4-PEG10K-NH_3_^+^	Polymer Factory

**Table 3:** List of the solvents.

Solvent	Acronym	Supplier Name
Dichloromethane	DCM	VWR International s.r.o (Stříbrná Skalice, Czech)
Formic acid	FA	VWR International s.r.o
Acetic acid	AA	VWR International s.r.o
Chloroform	CH	VWR International s.r.o
Methanol	Metha	VWR International s.r.o
Ethanol	Eth	VWR International s.r.o
Acetonitrile	Ace	VWR International s.r.o
Dimethylacetamide	DMAC	VWR International s.r.o
Dimethylformamide	DMF	VWR International s.r.o
Ethyl acetate	EA	VWR International s.r.o
Demineralized water	DW	-

**Table 4:** Process parameters of CA nanofibers electrospinning.

Parameters	Values	Units
Applied voltage	−20/+70	kV
Distance between electrodes	315	mm
Solution feeding rates	125	mbar/h
Solution feeding rates	100	mL/h
Nonwoven winding speed	1	mm/s
Humidity/Temperature	25/22	Rh%/°C

**Table 5:** Solution parameters, SEM images, and fiber diameters of CA nanofibers.

Polymer: Cellulose Acetate 398-10				
Main Solvents: DMAC/Ace				
Ratio of solvents	5/5	5/5	5/5	5/5
Solution concentration *w*/*v* (%)	10	12.5	15	17.5
SEM Image Magnifications 10k×–15k×–10k×	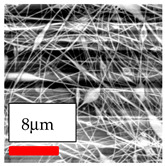	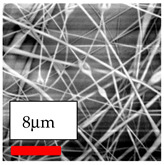	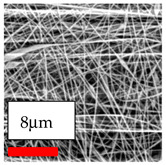	High viscosity no spinning
Fiber diameters (nm) ★ Best conditions	340 ± 35	410 ± 32	430 ± 42 ★	-
Ratio of solvents	9/1	7/3	3/7	1/9
Solution concentrations *w*/*v* (%)	15	15	15	15
SEM Image Magnifications 0–10k×–10k×	No fiber only solution spray—wet surface	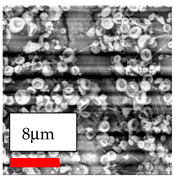	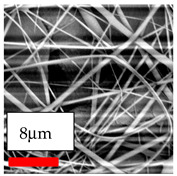	High viscosity No spinning
Fiber diameters (nm)	-	-	1375 ± 126	-

**Table 6:** Process parameters of PCL nanofibers electrospinning.

Parameters	Values	Units
Applied voltage	−30/+70	kV
Distance between electrodes	350	mm
Solution feeding rates	100–250	mbar/h
Solution feeding rates	100	mL/h
Nonwoven winding speed	1	mm/s
Humidity/Temperature	38/25	Rh%/°C

**Table 7:** Solution parameters, SEM images, and fiber diameters of PCL nanofibers.

Polymer: Polycaprolactone			
Main Solvents: Chloroform			
Solution concentration *w*/*v* (%)	10	12.5	15
Ratio of solvents	-	-	-
SEM Image Magnifications 5k×–1k×–1k×	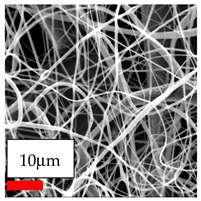	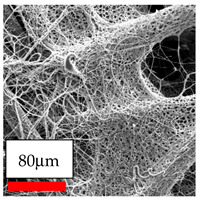	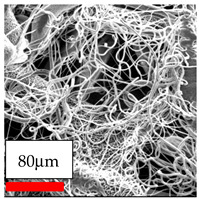
Fiber diameters (nm)	765 ± 152	1210 ± 134	1450 ± 164
Solution concentrations *w*/*v* (%)	10	12.5	15
Ratio of solvents	5/5 CH/Eth	5/5 CH/Eth	5/5 CH/Eth
SEM Image Magnifications 5k×–5k×–5k×	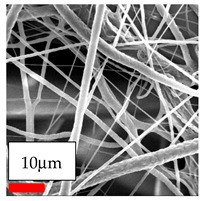	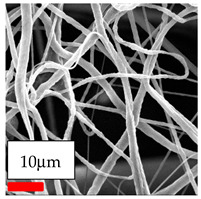	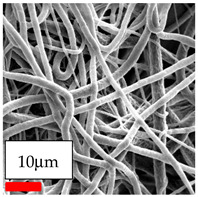
Fiber diameters (nm) ★ Best conditions	2430 ± 230	2870 ± 275	3055 ± 351 ★
Solution concentration *w*/*v* (%)	10	12.5	15
Ratio of solvents	5/5 CH/Metha	5/5 CH/Metha	5/5 CH/Metha
SEM Image Magnifications 5k×–5k×–5k×	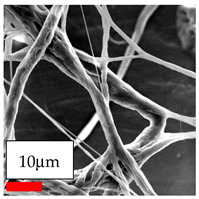	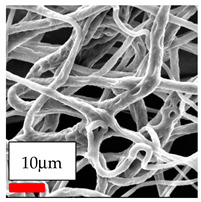	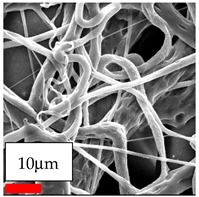
Fiber diameters (nm)	3240 ± 420	4330 ± 441	4680 ± 530
Solution concentrations *w*/*v* (%)	10	12.5	15
Ratio of solvents	5/5 CH/(AA:FA 1:1))	5/5 CH/(AA:FA 1:1)	5/5 CH/(AA:FA 1:1)
SEM Image Magnifications 500×–500×–1k×	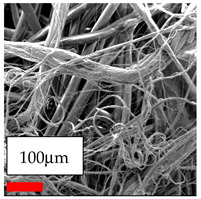	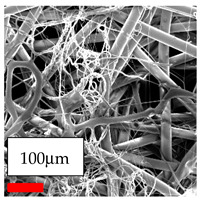	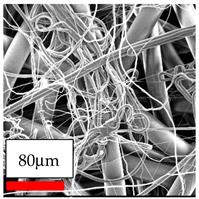
Fiber diameters (nm)	5430 ± 539	5670 ± 610	6055 ± 647
Solution concentration *w*/*v* (%)	10	12.5	15
Ratio of solvents	5/5 CH/Ace	5/5 CH/Ace	5/5 CH/Ace
SEM Image Magnifications 5k×–500×–5k×	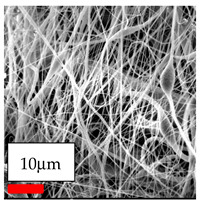	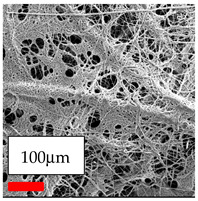	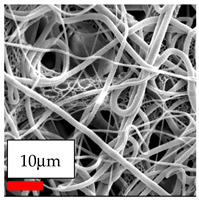
Fiber diameters (nm)	1890 ± 280	1975 ± 315	2055 ± 421
Solution concentration *w*/*v* (%)	10	12.5	15
Ratio of solvents	7/3 (AA:FA 1:1)/CH	7/3 (AA:FA 1:1)/CH	7/3 (AA:FA 1:1)/CH
SEM Image Magnifications 5k×–5k×–5k×	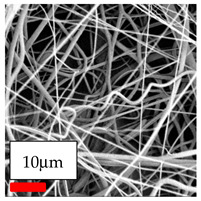	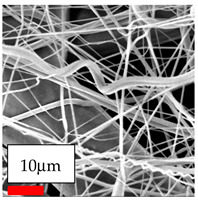	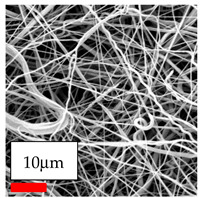
Fiber diameters (nm) ★ Best conditions	525 ± 95 ★	640 ± 110	670 ± 153
Solution concentration *w*/*v* (%)	15% (2/1) PCL/CA 398-3	15% (2/1) PCL/CAB CA 381-2	15% (2/1) PCL/CAP CA 482-0.5
Ratio of solvents	5/5 (AA:FA 1:1)/CH	5/5 (AA:FA1:1)/CH	5/5 (AA:FA 1:1)/CH
SEM Image Magnifications 5k×–5k×–5k×	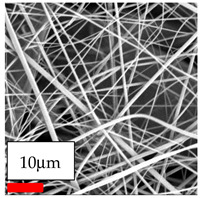	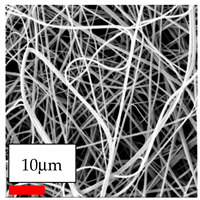	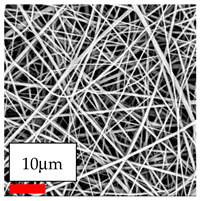
Fiber diameters (nm)	825 ± 168	840 ± 175	725 ± 158 ★
Solution concentration *w*/*v* (%)	15% (9/1) PCL/CS	15% (6/4) PCL/PEO	
Ratio of solvents	5/5 (AA:FA 1:1)/(CH:FA 1:1)	5/5 (AA:FA 1:1)/CH	
SEM Image Magnifications 10k×–5k×–0	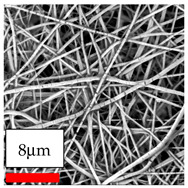	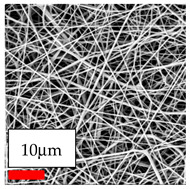	
Fiber diameters (nm) ★ Best conditions	775 ± 163 ★	670 ± 145 ★	

**Table 8:** Process parameters of PA6 nanofibers electrospinning.

Parameters	Values	Units
Applied voltage	−27/+70	kV
Distance between electrodes	320	mm
Solution feeding rates	80–100	mbar/h
Solution feeding rates	100	mL/h
Nonwoven winding speed	1	mm/s
Humidity/Temperature	35/22	Rh%/°C

**Table 9:** Solution parameters, SEM images, and fiber diameters of PA6 nanofibers.

**Polymer: Polyamide 6 (Econyl 27)**			
**Main Solvents: AA/FA**			
Solution concentration *w*/*v* (%)	10	12.5	15
Ratio of solvents	2/1	2/1	2/1
SEM Image Magnifications 30k×–20k×–20k×	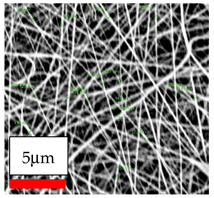	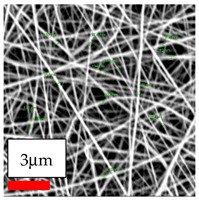	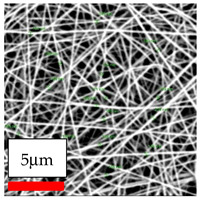
Fiber diameters (nm) ★ Best conditions	65 ± 29 ★	150 ± 53	200 ± 68
Solution concentration *w*/*v* (%)	10	12.5	15
Ratio of solvents	3/2	3/2	3/2
SEM Image Magnifications 10k×–20k×–10k×	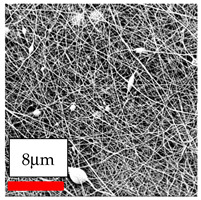	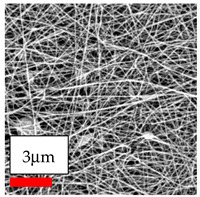	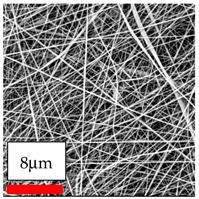
Fiber diameters (nm)	90 ± 35	135 ± 59	290 ± 83
Solution concentration *w*/*v* (%)	10	12.5	15
Ratio of solvents	1/1/1 AA/FA/DCM	1/1/1 AA/FA/DCM	1/1/1 AA/FA/DCM
SEM Image Magnifications 10k×–10k×–10k×	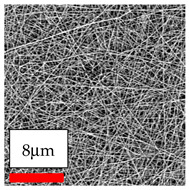	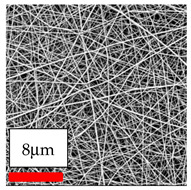	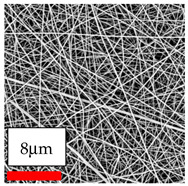
Fiber diameters (nm) ★ Best conditions	185 ± 63	440 ± 84 ★	620 ± 126
Solution concentration *w*/*v* (%)	10	12.5	15
Ratio of solvents	1/1/1 AA/FA/CH	1/1/1 AA/FA/CH	1/1/1 AA/FA/CH
SEM Image Magnifications 10k×–10k×–5k×	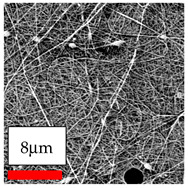	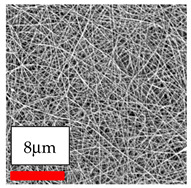	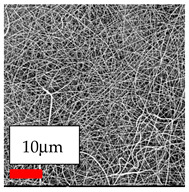
Fiber diameters (nm) ★ Best conditions	130 ± 75	230 ± 80 ★	520 ± 113 ★
Polymer: Polyamide 6 (BASF 24)			
Main solvents: AA/FA			
Solution concentration *w*/*v* (%)	10	12.5	15
Ratio of solvents	1/1/1 AA/FA/DCM	1/1/1 AA/FA/DCM	1/1/1 AA/FA/DCM
SEM Image Magnifications 20k×–20k×–20k×	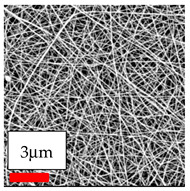	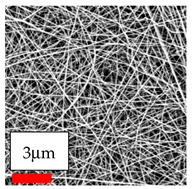	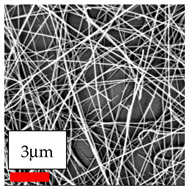
Fiber diameters (nm) ★ Best conditions	220 ± 135	310 ± 142 ★	400 ± 168
Solution concentration *w*/*v* (%)	10	12.5	15
Ratio of solvents	1/1/1 AA/FA/CH	1/1/1 AA/FA/CH	1/1/1 AA/FA/CH
SEM Image Magnifications 10k×–20k×–10k×	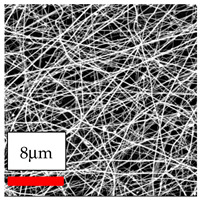	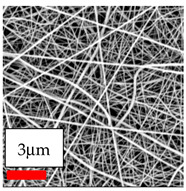	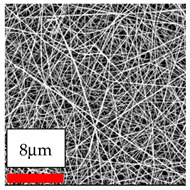
Fiber diameters (nm) ★ Best conditions	250 ± 95	400 ± 148 ★	650 ± 163 ★

**Table 10:** Process parameters of PA11 and PA12 nanofibers electrospinning.

Parameters	Values	Units
Applied voltage	−30/+70	kV
Distance between electrodes	315	mm
Solution feeding rates	80–100	mbar/h
Solution feeding rates	100	mL/h
Nonwoven winding speed	1	mm/s
Humidity/Temperature	30/28	Rh%/°C

**Table 11:** Solution parameters, SEM images, and fiber diameters of PA11, PA12 nanofibers.

Polymer: Polyamide 11, 12, and Polyvinyl Butyral			
Main Solvents: FA/DCM			
Polymer: Polyamide 11			
Solution concen. *w*/*v* (%)	10	12.5	15
Ratio of solvents	1/1 FA/DCM	1/1 FA/DCM	1/1 FA/DCM
SEM Image Magnifications 10k×–20k×–10k×	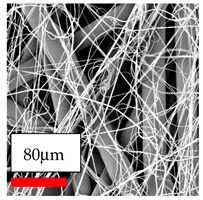	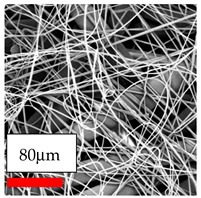	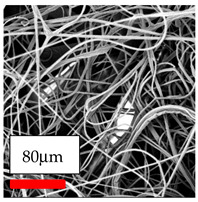
Fiber diameters (nm)	4590 ± 463	6455 ± 519	6745 ± 736
Polymer: Polyamide 12			
Main solvents: FA/DCM			
Solution concen. *w*/*v* (%)	10	12.5	10
Ratio of solvents	1/1 FA/DCM	1/1 FA/DCM	FA
SEM Image Magnifications 5k×–1k×–1k×	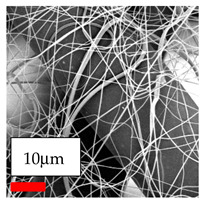	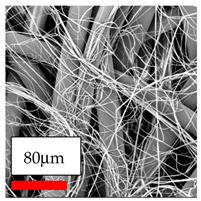	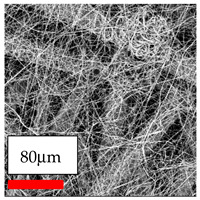
Fiber diameters (nm) ★ Best conditions	620 ± 475	1465 ± 498	790 ± 517 ★
Solution concen. *w*/*v* (%)	12.5	15 (2/1) PA11/ PVB	15 (2/1) PA12/ PVB
Ratio of solvents	FA	1/1 FA/DCM	1/1 FA/DCM
SEM Image Magnifications 1k×–2.5k×–5k×	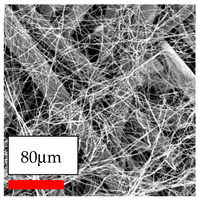	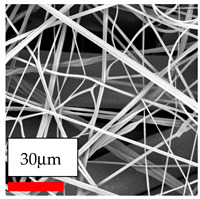	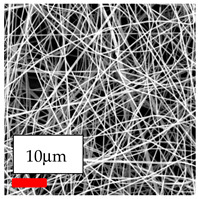
Fiber diameters (nm) ★ Best conditions	1510 ± 264	2395 ± 454	870 ± 224 ★

**Table 12:** Process parameters of PAN nanofibers electrospinning.

Parameters	Values	Units
Applied voltage	−30/+70	kV
Distance between electrodes	350	mm
Solution feeding rates	100–150	mbar/h
Solution feeding rates	100	mL/h
Nonwoven winding speed	1	mm/s
Humidity/Temperature	21/23	Rh%/°C

**Table 13:** Solution parameters, SEM images and fiber diameters of PAN nanofibers.

Polymer: Polyacrylonitrile			
Main Solvents: DMAC and DMF			
Solution concen. *w*/*v* (%)	9	10	12.5
Ratio of solvents	DMAC	DMAC	DMAC
SEM Image Magnifications 10k×–10k×–10k×	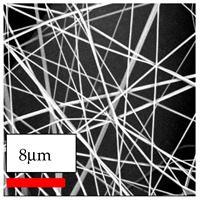	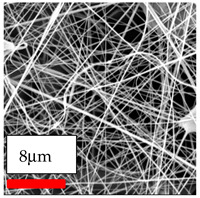	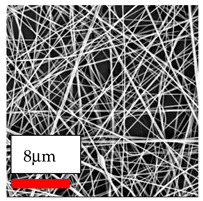
Fiber diameters (nm)	335 ± 132	350 ± 146	410 ± 155
Solution concen. *w*/*v* (%)	10	12.5	15
Ratio of solvents	DMF	DMF	DMF
SEM Image Magnifications 10k×–10k×–10k×	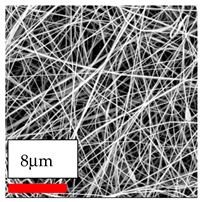	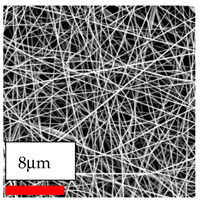	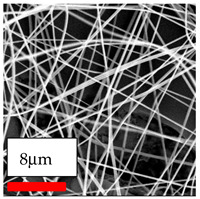
Fiber diameters (nm) ★ Best conditions	165 ± 94	210 ± 106 ★	545 ± 210 ★
Solution concen. *w*/*v* (%)	17.5	20	
Ratio of solvents	DMF	DMF	
SEM Image Magnifications 10k×–5k×	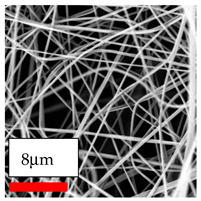	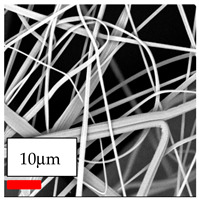	
Fiber diameters (nm)	720 ± 258	2670 ± 410	

**Table 14:** Process parameters of PVDF nanofibers electrospinning.

Parameters	Values	Units
Applied voltage	−30/+70	kV
Distance between electrodes	320	mm
Solution feeding rates	50–150	mbar/h
Solution feeding rates	100	mL/h
Nonwoven winding speed	1	mm/s
Humidity/Temperature	24/20	Rh%/°C

**Table 15:** Solution parameters, SEM images, and fiber diameters of PVDF nanofibers.

Polymer: Polyvinylidene Fluoride			
Main Solvents: DMAC + 3%TEAB in DMF			
Solution concen. *w*/*v* (%)	10	12.5	15
Ratio of solvents	50/1 DMAC/TEAB in DMF	50/1 DMAC/TEAB in DMF	50/1 DMAC/TEAB in DMF
SEM Image Magnifications 10k×–10k×–10k×	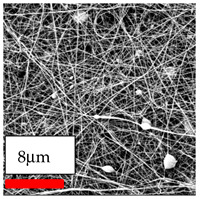	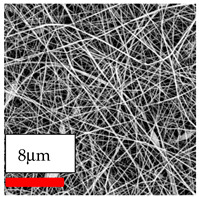	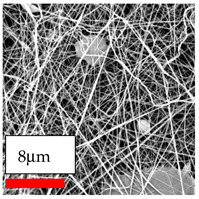
Fiber diameters (nm)	225 ± 124	325 ± 167	440 ± 189
Solution concen. *w*/*v* (%)	17.5	20	
Ratio of solvents	50/1 DMAC/TEAB in DMF	50/1 DMAC/TEAB in DMF	
SEM Image Magnifications 10k×–10k×	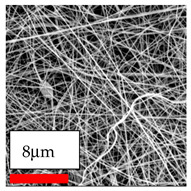	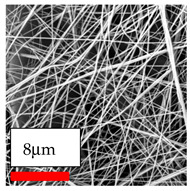	
Fiber diameters (nm) ★ Best conditions	495 ± 185 ★	590 ± 210	

**Table 16:** Process parameters of PU nanofibers electrospinning.

Parameters	Values	Units
Applied voltage	−30/+70	kV
Distance between electrodes	320	mm
Solution feeding rates	100–200	mbar/h
Solution feeding rates	100	mL/h
Nonwoven winding speed	1	mm/s
Humidity/Temperature	28/22	Rh%/°C

**Table 17:** Solution parameters, SEM images, and fiber diameters of PU nanofibers.

Polymer: Polyurethane			
Main Solvents: DMF + 3%TEAB in DMF			
Solution concen. *w*/*v* (%)	10	15	20
Ratio of solvents	DMF	DMF	DMF
SEM Image Magnifications 5k×–5k×–5k×	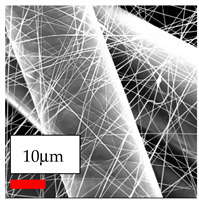	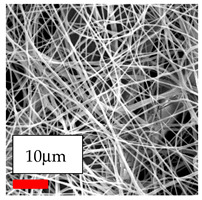	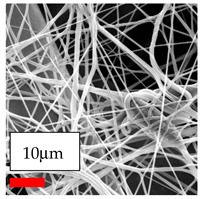
Fiber diameters (nm)	445 ± 138	680 ± 165	1410 ± 215
Solution concen. *w*/*v* (%)	10	12.5	15
Ratio of solvents	DMF/TEAB in DMF 50/1	DMF/TEAB in DMF 50/1	DMF/TEAB in DMF 50/1
SEM Image Magnifications 5k×–10k×–10k×	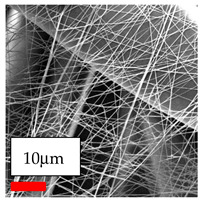	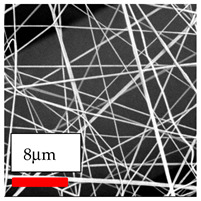	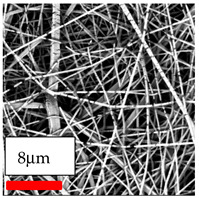
Fiber diameters (nm) ★ Best conditions	470 ± 174	560 ± 186	665 ± 242 ★
Solution concen. *w*/*v* (%)	17.5	15 PU/PVB 10/1	
Ratio of solvents	DMF/TEAB in DMF 50/1	DMF	
SEM Image Magnifications 10k×–5k×	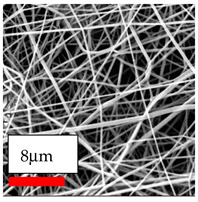	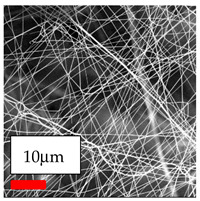	
Fiber diameters (nm) ★ Best conditions	720 ± 255 ★	450 ± 195	

**Table 18:** Process parameters of PVB nanofibers electrospinning.

Parameters	Values	Units
Applied voltage	−25/+70	kV
Distance between electrodes	290	Mm
Solution feeding rates	80–120	mbar/h
Solution feeding rates	100	mL/h
Nonwoven winding speed	1	mm/s
Humidity/Temperature	30/25	Rh%/°C

**Table 19:** Solution parameters, SEM images, and fiber diameters of PVB nanofibers.

Polymer: Polyvinyl Butyral			
Main Solvents: Ethanol			
Solution concen. *w*/*v* (%)	10	12.5	15
Ratio of solvents	1/1 EtOH/CH	1/1 EtOH/CH	1/1 EtOH/CH
SEM Image Magnifications 5k×–5k×–	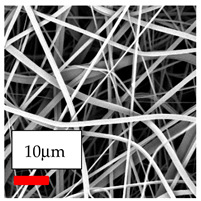	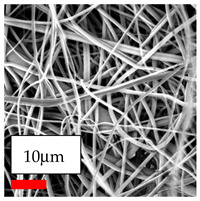	Too viscous polymer solution—no fibers
Fiber diameters (nm)	1455 ± 358	1580 ± 377	
Solution concen. *w*/*v* (%)	10	12.5	15
Ratio of solvents	1/1 EtOH/(AA:FA)	1/1 EtOH/(AA:FA)	1/1 EtOH/(AA:FA)
SEM Image Magnifications 5k×–10k×–5k×	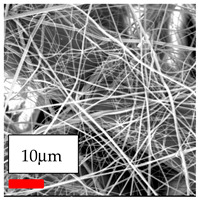	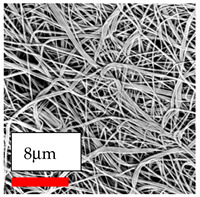	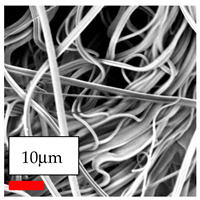
Fiber diameters (nm) ★ Best conditions	720 ± 203 ★	890 ± 274	2640 ± 298
Solution concen. *w*/*v* (%)	10	12.5	15
Ratio of solvents	1/1 EtOH/EtAC	1/1 EtOH/EtAC	1/1 EtOH/EtAC
SEM Image Magnifications 10k×–5k×–5k×	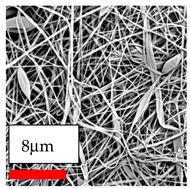	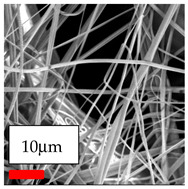	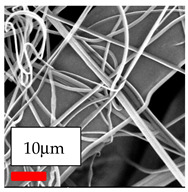
Fiber diameters (nm)	510 ± 165	1630 ± 248	2150 ± 313

**Table 20:** Process parameters of PVA nanofibers electrospinning.

Parameters	Values	Units
Applied voltage	−25/+70	kV
Distance between electrodes	350	mm
Solution feeding rates	100–250	mbar/h
Solution feeding rates	100	mL/h
Nonwoven winding speed	1	mm/s
Humidity/Temperature	20/30	Rh%/°C

**Table 21:** Solution parameters, SEM images, and fiber diameters of PVA nanofibers.

Polymer: Polyvinyl Alcohol			
Main Solvents: DW, EtOH, AA/FA (1/1), DMF			
Solution concen. *w*/*v* (%)	10	12	14
Ratio of solvents	Demi-water	Demi-water	Demi-water
SEM Image Magnifications 1k×–10k×–5k×	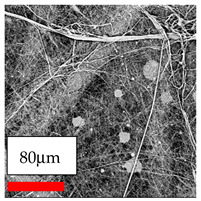	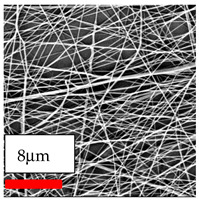	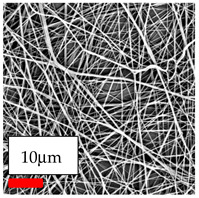
Fiber diameters (nm) ★ Best conditions	360 ± 103	435 ± 152 ★	690 ± 194
Solution concen. *w*/*v* (%)	10	12	14
Ratio of solvents	EtOH	EtOH	EtOH
SEM Image Magnifications 5k×–5k×–5k×	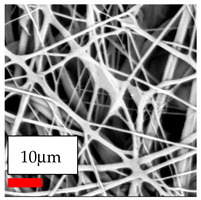	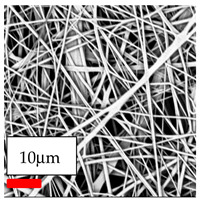	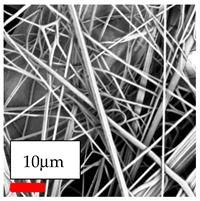
Fiber diameters (nm)	1160 ± 234	1280 ± 253	2430 ± 279
Solution concen. *w*/*v* (%)	10	12	14
Ratio of solvents	AA/FA (1/1)	AA/FA (1/1)	AA/FA (1/1)
SEM Image Magnifications –20k×–5k×	Low viscosity, wet surface	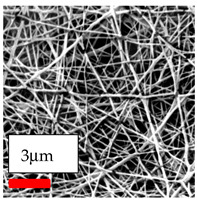	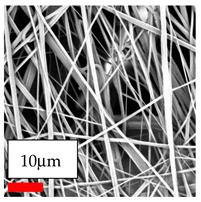
Fiber diameters (nm) ★ Best conditions		245 ± 87 ★	1965 ± 268
Solution concen. *w*/*v* (%)	10	12	14
Ratio of solvents	DMF	DMF	DMF
SEM Image Magnifications –10k×–5k×	Low viscosity, wet surface	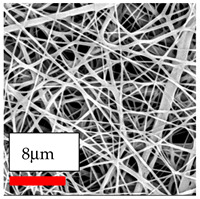	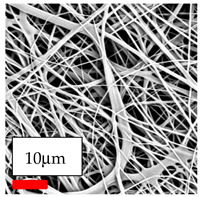
Fiber diameters (nm)		570 ± 164	1090 ± 236

**Table 22:** Process parameters of PA6 nanofibers containing nanoparticles during electrospinning.

Parameters	Values	Units
Applied voltage	−27/+70	kV
Distance between electrodes	320	mm
Solution feeding rates	80–100	mbar/h
Solution feeding rates	100	mL/h
Nonwoven winding speed	1	mm/s
Humidity/Temperature	35/22	Rh%/°C

**Table 23:** Solution parameters, SEM images of PA6 nanofibers containing nanoparticles.

Polymer: 15 *w*/*v* % PA6			
Main Solvents and Ratio: AA/FA/CH 1/1/1			
Type of Nanoparticle	TiO_2_ 200 nm		
Nanoparticle concen. w (%)	5	15	30
SEM Images Magnifications 20k×–20k×–20k×	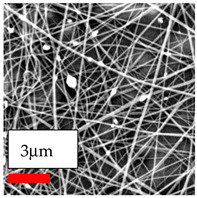	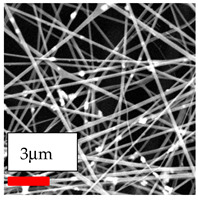	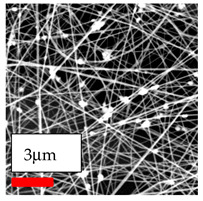
Type of Nanoparticle	ZnO 30–50 nm		
Nanoparticle concen. w (%)	5	15	30
SEM Images Magnifications 20k×–10k×–20k×	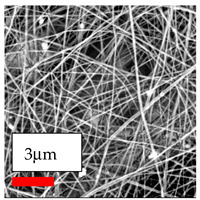	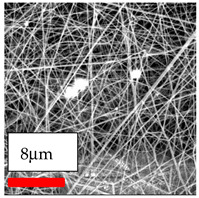	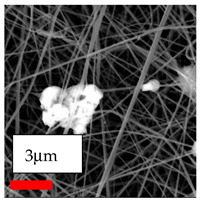
Type of Nanoparticle	MgO 55 nm		
Nanoparticle concen. w (%)	5	15	30
SEM Images Magnifications 10k×–10k×–10k×	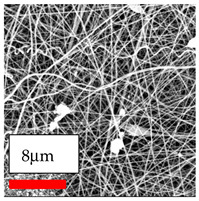	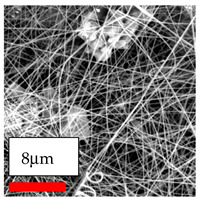	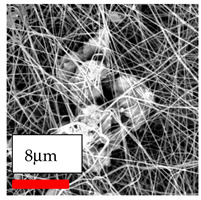
Type of Nanoparticle	MgO 500–1500 nm		
Nanoparticle concen. w (%)	5	15	30
SEM Images Magnifications 10k×–5k×–10k×	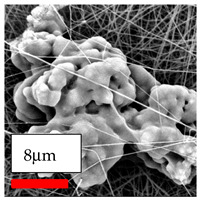	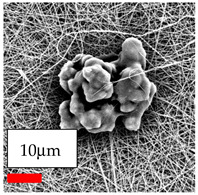	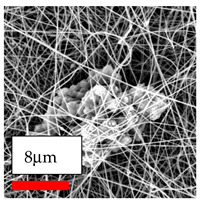
Type of Nanoparticle	CuO 38 nm		
Nanoparticle concen. w (%)	5	15	30
SEM Images Magnifications 10k×–5k×–5k×	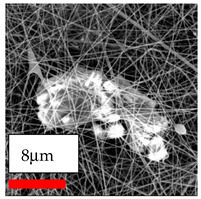	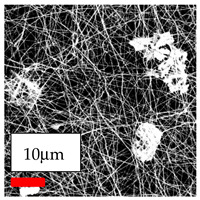	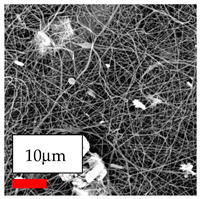
Type of Nanoparticle	CuO 78 nm		
Nanoparticle concen. w (%)	5	15	30
SEM Images Magnifications 10k×–5k×–5k×	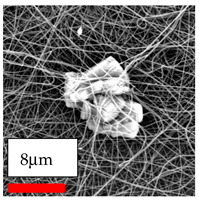	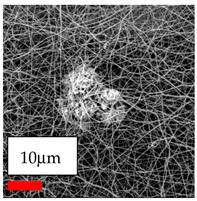	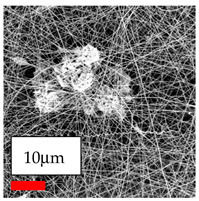
Type of Nanoparticle	CuO 20 micron		
Nanoparticle concen. w (%)	5	15	30
SEM Images Magnifications 5k×–5k×–5k×	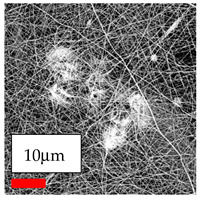	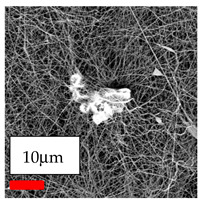	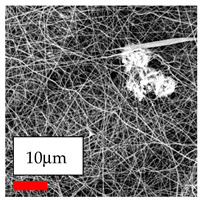
Type of Nanoparticle	Ag		
Nanoparticle concen. w (%)	5	15	30
SEM Images Magnifications 10k×–5k×–10k×	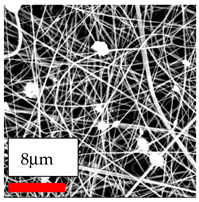	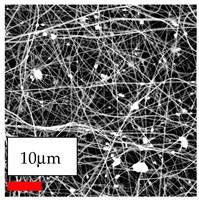	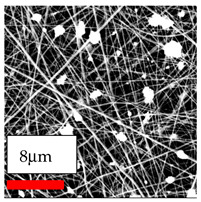
Type of Nanoparticle	Graphene Oxide		
Nanoparticle concen. w (%)	5	15	30
SEM Images Magnifications 5k×–10k×–10k×	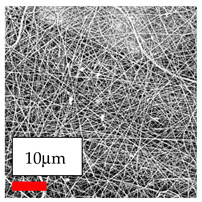	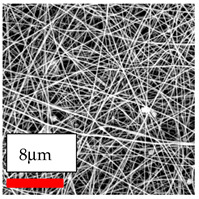	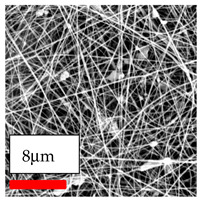
Type of Nanoparticle	CeO_2_		
Nanoparticle concen. w (%)		15	30
SEM Images Magnifications –5k×–5k×		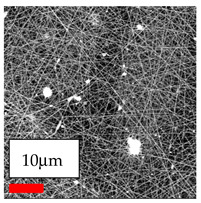	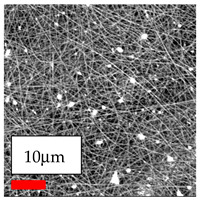
Type of Nanoparticle	CeO_2_/TiO_2_		
Nanoparticle concen. w (%)	(15/15)	(30/15)	(15/30)
SEM Images Magnifications 15k×–10k×–25k×	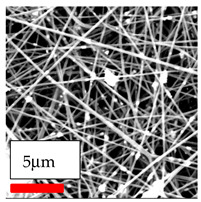	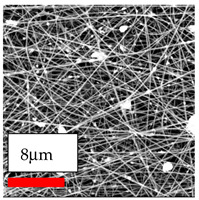	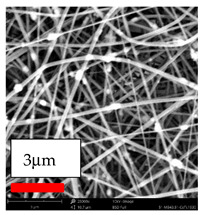
Type of Nanoparticle	CeO_2_/ZnO		
Nanoparticle concen. w (%)	(15/15)	(30/15)	(15/30)
SEM Images Magnifications 10k×–10k×–10k×	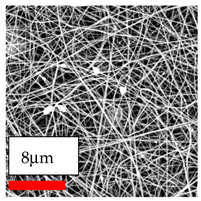	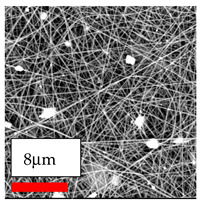	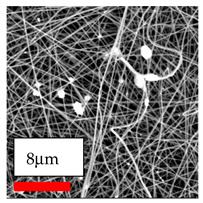
Type of Nanoparticle	Er_2_O_3_		
Nanoparticle concen. w (%)		15	30
SEM Images Magnifications –10k×–10k×		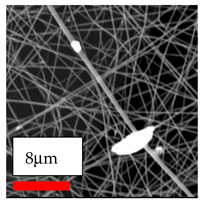	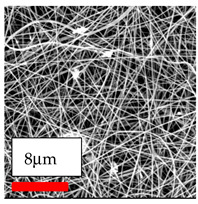
Type of Nanoparticle	Er_2_O_3_/TiO_2_		
Nanoparticle concen. w (%)	(15/15)	(30/15)	(15/30)
SEM Images Magnifications 20k×–20k×–20k×	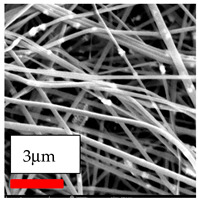	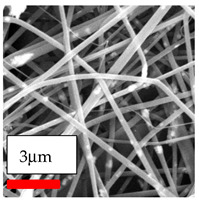	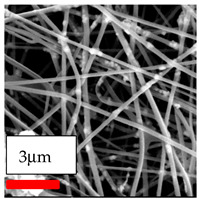
Type of Nanoparticle	Er_2_O_3_/ZnO		
Nanoparticle concen. w (%)	(15/15)	(30/15)	(15/30)
SEM Images Magnifications 10k×–10k×–10k×	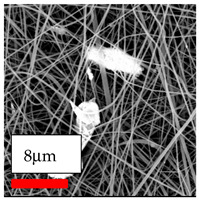	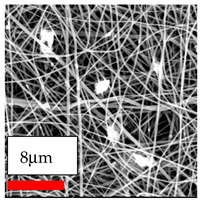	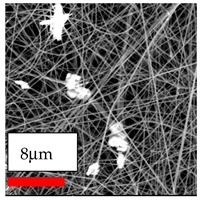
Type of Nanoparticle	WO_3_		
Nanoparticle concen. w (%)		15	30
SEM Images Magnifications –10k×–10k×		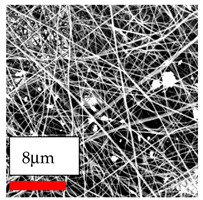	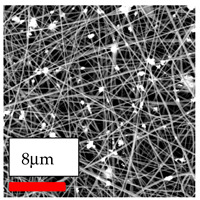
Type of Nanoparticle	WO_3_/TiO_2_		
Nanoparticle concen. w (%)	(15/15)	(30/15)	(15/30)
SEM Images Magnifications 20k×–20k×–10k×	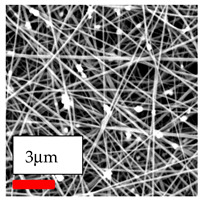	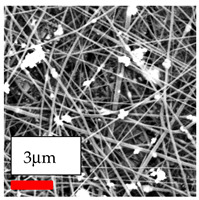	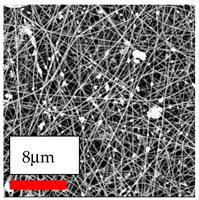
Type of Nanoparticle	WO_3_/ZnO		
Nanoparticle concen. w (%)	(15/15)	(30/15)	(15/30)
SEM Images Magnifications 10k×–10k×–10k×	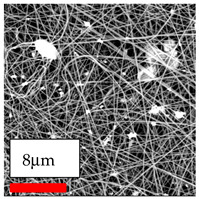	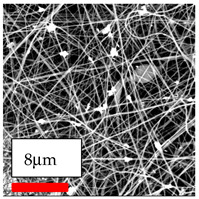	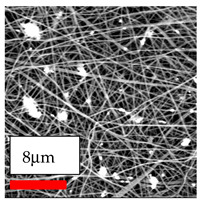
Type of Nanoparticle	MnO_2_		
Nanoparticle concen. w (%)		15	30
SEM Images Magnifications –10k×–10k×		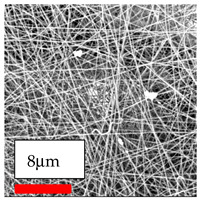	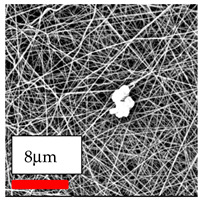
Type of Nanoparticle	MnO_2_/GO		
Nanoparticle concen. w (%)	(15/15)	(30/15)	(15/30)
SEM Images Magnifications 10k×–10k×–10k×	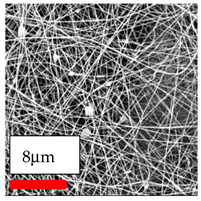	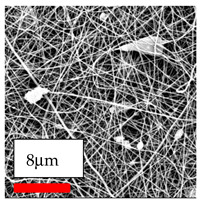	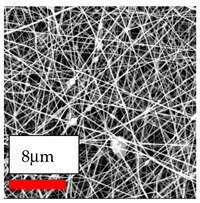
Type of Nanoparticle	TiO_2_/GO		
Nanoparticle concen. w (%)	(15/15)	(30/15)	(15/30)
SEM Images Magnifications 20k×–10k×–10k×	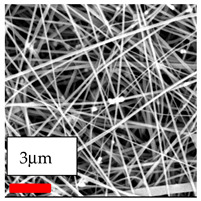	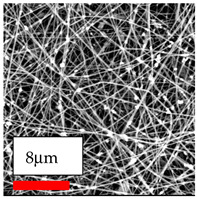	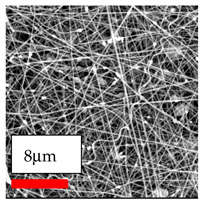
Type of Nanoparticle	CeO_2_/GO		
Nanoparticle concen. w (%)	(15/15)	(30/15)	(15/30)
SEM Images Magnifications 10k×–10k×–10k×	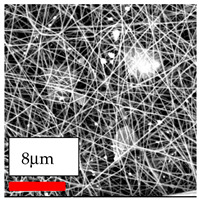	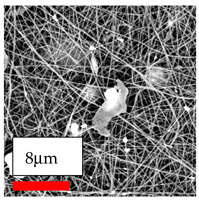	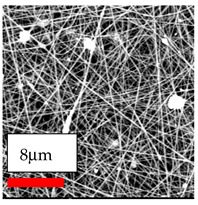
Type of Nanoparticle	WO_3_/GO		
Nanoparticle concen. w (%)	(15/15)	(30/15)	(15/30)
SEM Images Magnifications 10k×–10k×–10k×	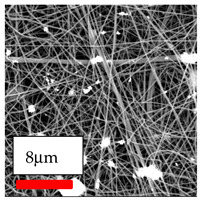	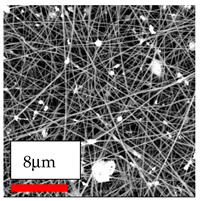	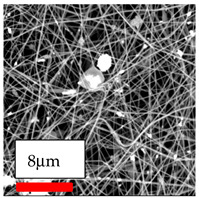
Type of Nanoparticle	Er_2_O_3_/GO		
Nanoparticle concen. w (%)	(15/15)	(30/15)	(15/30)
SEM Images Magnifications 10k×–10k×–10k×	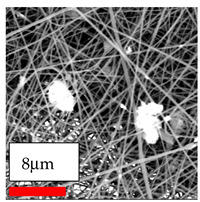	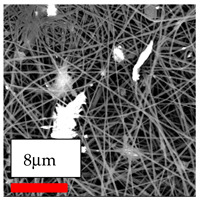	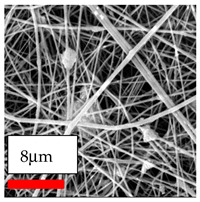
Type of Nanoparticle	Hyperbranched polymer		
Nanoparticle concen. w (%)	1% HBPG4-OH	1% HBPG4-NH	
SEM Images Magnifications 10k×–10k×–	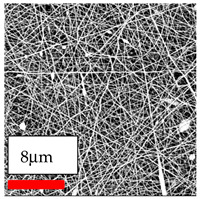	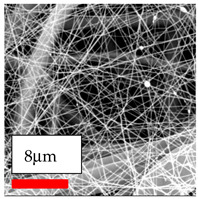	

**Table 24:** Process parameters of PAN nanofibers containing nanoparticles during electrospinning.

Parameters	Values	Units
Applied voltage	−30/+70	kV
Distance between electrodes	350	mm
Solution feeding rates	100–150	mbar/h
Solution feeding rates	100	mL/h
Nonwoven winding speed	1	mm/s
Humidity/Temperature	21/23	Rh%/°C

**Table 25:** Solution parameters, SEM images of PAN nanofibers containing nanoparticles.

Polymer: 15 *w*/*v* % PAN			
Main Solvents and Ratio: DMF			
Type of Nanoparticle	TiO_2_ NP—200 nm	ZnO—30–50 nm	MgO NP—55 nm
Nanoparticle concen. w (%)	15	15	15
SEM Images	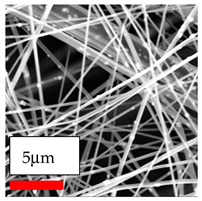	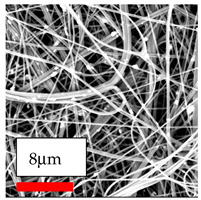	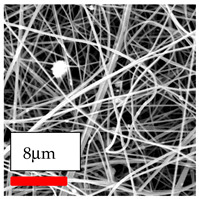
Type of Nanoparticle	MgO/TiO_2_ NP	MgO/ZnO	
Nanoparticle concen. w (%)	15/15	15/15	
SEM Images	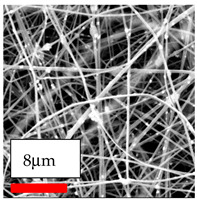	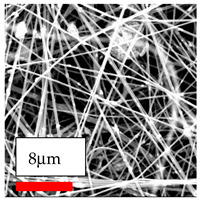	
Type of Nanoparticle	CeO_2_	CeO_2_/TiO_2_	CeO_2_/ZnO
Nanoparticle concen. w (%)	15	15/15	15/15
SEM Images	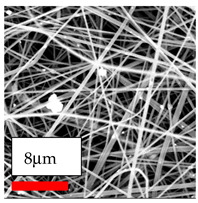	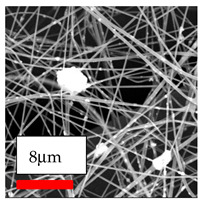	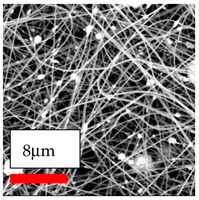
Type of Nanoparticle	Er_2_O_3_	Er_2_O_3_/TiO_2_	Er_2_O_3_/ZnO
Nanoparticle concen. w (%)	15	15/15	15/15
SEM Images	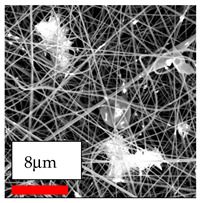	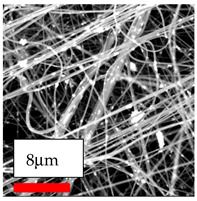	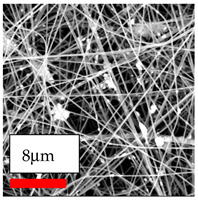
Type of Nanoparticle	WO_3_	WO_3_/TiO_2_	WO_3_/ZnO
Nanoparticle concen. w (%)	15	15/15	15/15
SEM Images	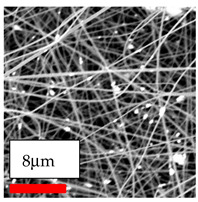	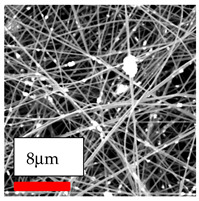	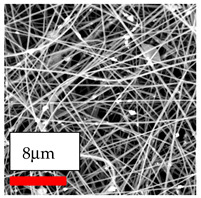
Type of Nanoparticle	MnO_2_	MnO_2_/TiO_2_	MnO_2_/ZnO
Nanoparticle concen. w (%)	15	15/15	15/15
SEM Images	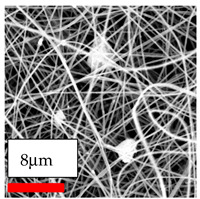	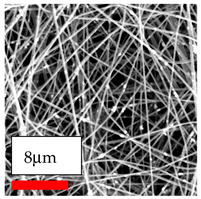	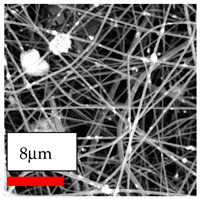
Type of Nanoparticle	GO	GO/TiO_2_	GO/ZnO
Nanoparticle concen. w (%)	15	15/15	15/15
SEM Images	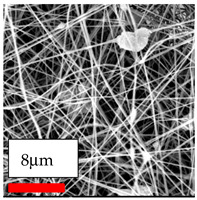	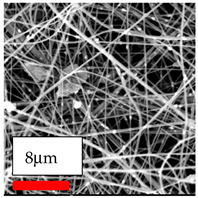	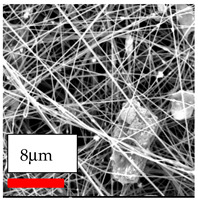

**Table 26:** Process parameters of PU nanofibers containing nanoparticles during electrospinning.

Parameters	Values	Units
Applied voltage	−30/+70	kV
Distance between electrodes	350	mm
Solution feeding rates	100–150	mbar/h
Solution feeding rates	100	mL/h
Nonwoven winding speed	1	mm/s
Humidity/Temperature	21/23	Rh%/°C

**Table 27:** Solution parameters, SEM images of PU nanofibers containing nanoparticles.

Polymer: 14.5 *w*/*v* % PU			
Main Solvents and Ratio: DMF			
Type of Nanoparticle	TiO_2_ NP—200 nm	ZnO—30–50 nm	MgO NP—55 nm
Nanoparticle concen. w (%)	15	15	15
SEM Images	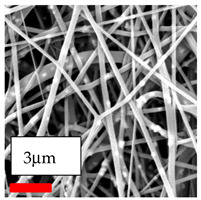	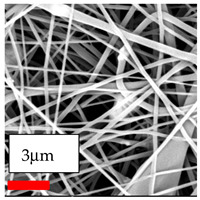	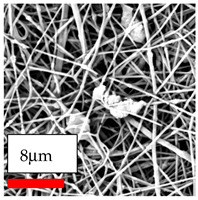
Type of Nanoparticle	MgO/TiO_2_ NP	MgO/ZnO	
Nanoparticle concen. w (%)	15/15	15/15	
SEM Images	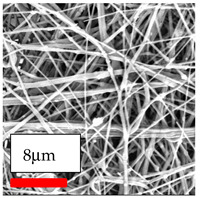	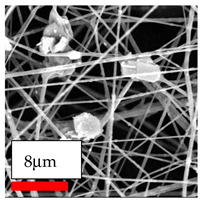	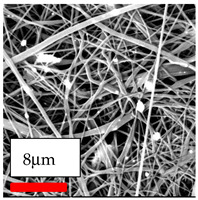
Type of Nanoparticle	CeO_2_	CeO_2_/TiO_2_	CeO_2_/ZnO
Nanoparticle concen. w (%)	15	15/15	15/15
SEM Images	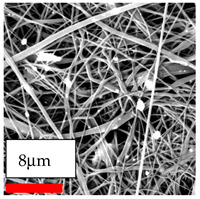	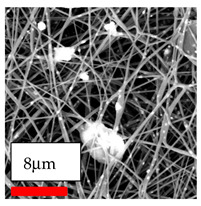	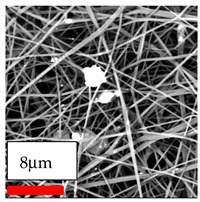
Type of Nanoparticle	WO_3_	WO_3_/TiO_2_	WO_3_/ZnO
Nanoparticle concen. w (%)	15	15/15	15/15
SEM Images	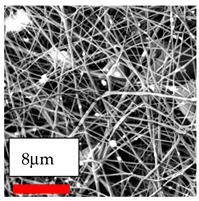	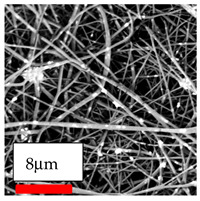	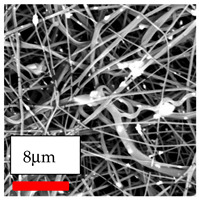
Type of Nanoparticle	MnO_2_	MnO_2_/TiO_2_	MnO_2_/ZnO
Nanoparticle concen. w (%)	15	15/15	15/15
SEM Images	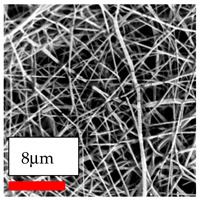	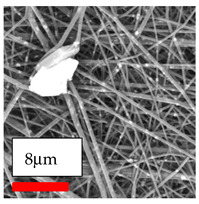	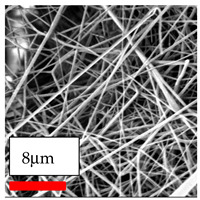
Type of Nanoparticle	GrO	GrO/TiO_2_	GrO/ZnO
Nanoparticle concen. w (%)	15	15/15	15/15
SEM Images	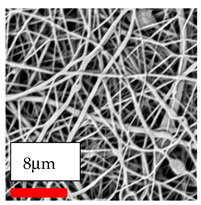	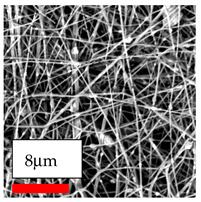	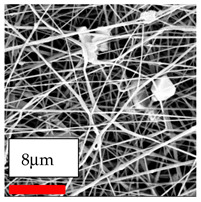

**Table 28:** Applications of all the mentioned polymeric nanofibers and their summary.

Polymer	Applications of Nanofibers	Summary
PA6	Filtration membranes [112], sportwear and protective textiles [17], biomedical scaffolds [113], air and water purification [114]	Widely used in filtration thanks to the small fiber diameter.
PA11	Biomedical devices, sensors [115], membrane separation, eco-friendly engineering applications [116]	Bio-based polymer for sustainable applications.
PA12	Oil-water separation membranes, biomedical materials	Industrial filters due to low water absorption.
PVB	Air filtration [117], battery separators [118], sound-insulating materials [119]	Versatile and flexible nanofibers.
PCL	Tissue engineering scaffolds [60], drug delivery systems [61], wound healing applications [82]	Medical applications, thanks to biodegradability.
PAN	Filtration membranes [120], energy storage [121](supercapacitors), carbon nanofiber precursors [122], protective textiles [107]	Excellent thermal stability and chemical resistance for challenging applications.
PVDF	Battery separators [123], piezoelectric sensors [124,125], water treatment membranes [126], energy harvesting devices [127], food packaging [128]	Excellent weathering and chemical resistance.
PU	Wound dressings [129], biomedical scaffolds [130], breathable protective clothing [131], filtration membranes [132]	Flexible, elastic, suitable for apparel and wound healing.
PVA	Biomedical applications [133] (drug delivery, wound healing), food packaging [134], water filtration membranes [135]	hydrophilic, biodegradable, and non-toxic, widely used in biomedical fields.
CA	Air and water filtration [136], biodegradable packaging [137], drug delivery systems [138], membrane separation [139]	Exhibit excellent biocompatibility and biodegradability.
PA6/NP	Antibacterial textiles [140], photocatalytic membranes [141], self-cleaning surfaces [142], heavy metal removal from wastewater [143]	Enhances antibacterial, photocatalytic, and self-cleaning properties
PAN/NP	Filtration membranes [144], antibacterial materials [145], enhanced adsorption capacity [146], UV protection [147]	Provides enhanced photocatalytic activity, antibacterial behavior, and pollutant adsorption
PU/NP	Antimicrobial wound dressings [148], UV-resistant coatings [149], smart textiles [150], gas sensors [151]	Provides antimicrobial activity, UV resistance

## Data Availability

Data are contained within the article.
